# Identification of MicroRNAs Regulating *Clostridium perfringens* Type C Infection in the Spleen of Diarrheic Piglets

**DOI:** 10.3390/cimb45040208

**Published:** 2023-04-06

**Authors:** Pengfei Wang, Qiaoli Yang, Zunqiang Yan, Xiaoyu Huang, Xiaoli Gao, Shuangbao Gun

**Affiliations:** 1 College of Animal Science and Technology, Gansu Agricultural University, Lanzhou 730070, China; 2 Gansu Research Center for Swine Production Engineering and Technology, Gansu Agricultural University, Lanzhou 730070, China

**Keywords:** piglet, diarrhea, spleen, miRNA, *C. perfringens* type C, resistance

## Abstract

*Clostridium perfringens* (*C. perfringens*) type C is one of the common bacteria in piglet diarrhea, which seriously affects the swine industry’s development. The spleen plays crucial roles in the resistance and elimination of pathogenic microorganisms, and miRNAs play important roles in regulating piglet diarrhea caused by pathogens. However, the mechanism by which miRNAs in the spleen are involved in regulating *C. perfringens* type C causing diarrhea in piglets remains unclear. The expression profiles of the spleen miRNAs of 7-day-old piglets challenged by *C. perfringens* type C were studied using small RNA-sequencing in control (SC), susceptible (SS), and resistant (SR) groups. Eight-eight differentially expressed miRNAs were screened. The KEGG pathway analysis of target genes revealed that the miRNAs were involved in the MAPK, p53, and ECM–receptor interaction signaling pathways. *NFATC4* was determined to be a direct target of miR-532-3p and miR-133b using a dual-luciferase reporter assay. Thus, miR-133b and miR-532-3p targeted to *NFATC4* were likely involved to piglet resistance to *C. perfringens* type C. This paper provides the valuable resources to deeply understand the genetic basis of *C. perfringens* type C resistance in piglets and a solid foundation to identify novel markers of *C. perfringens* type C resistance.

## 1. Introduction

Diarrhea is a common symptom of infectious intestinal diseases, leading to death of piglets. These diseases have caused great economic losses to the pig industry and has become a stumbling block to the healthy development of the pig industry.

*C. perfringens* type C, a spore-forming bacillus, is one of the common pathogenic bacteria in piglets associated with bacterial diarrhea [1,2]. Piglets are infected through the digestive tract, and the bacteria largely propagate in the intestinal tract. The resulting toxins destroy intestinal tissues and travel through the humoral circulatory system to damage distal organs such as the spleen, causing diarrhea and systemic organ damage in piglets. The spleen is composed of white pulp, red pulp, and marginal area, and the unique tissue structure of the spleen is the foundation for its resistance to pathogen infections [3,4]. Infectious diseases often lead to atrophy or enlargement of the spleen, thus endangering the health of the organism. Moreover, the incidence of infectious diseases increases substantially after spleen removal, demonstrating its crucial role in immune regulation in the resistance and elimination of pathogenic microorganisms [5,6]. Previous studies showed that the spleen plays an important role in resisting *C. perfringens* infections. Dinh et al. and Anh et al. showed that White Leghorn chickens reduce food intake and experience weight loss, intestinal mucosa edema, and enlarged spleens after drenching with 1 × 10^9^ colony-forming unit (CFU)/mL *C. perfringens* type A [7,8]. Similarly, Hong et al. reported that Ross chickens showed spleen enlargement, mucosal edema, and diarrhea after drenching with 1 × 10^9^ CFU/mL *C. perfringens* type A [9]. In those studies, the spleen produced an immune response after being challenged with *C. perfringens* type A.

MicroRNAs (miRNAs) are single-stranded, endogenous, highly conserved, non-coding RNA (ncRNA) small molecules, containing approximately 21 to 23 nucleotides, in eukaryotes [10,11]. These miRNAs cause translational repression or deadenylation of target mRNAs via binding to complementary target sites in the 3′ UTR of mRNA [12,13]. miRNAs regulate gene expression at the post-transcriptional level by encoding target protein mRNAs that participate in cellular development, proliferation, apoptosis, virus defense, fat metabolism, and immune response [14,15]. miRNAs are involved in the regulation of chicken necrotic enteritis caused by *C. perfringens* type A infection. Hong et al. reported that *TGFβR2* gene expression was correlated with altered gga-miR-216 levels in Marek’s disease-susceptible White Leghorn chickens and indicated that the miRNA played key regulatory roles in diarrheic chickens infected with *C. perfringens* type A [16].

Recently, the authors of this paper studied the miRNAs in the ileum of piglets with diarrhea caused by *C. perfringens* type C and screened three miRNAs that likely assisted in resisting *C. perfringens* type C infection [17]. However, the mechanism by which miRNAs in the spleen are involved in regulating *C. perfringens* type C-induced diarrhea in piglets remains unclear. Therefore, identification of the miRNAs involved in the regulation of piglet resistance to diarrhea and exploration of their regulatory mechanisms will greatly increase our understanding of the genetic mechanisms of piglet resistance to diarrhea caused by *C. perfringens* type C infection.

This study aimed to explore the molecules related to diarrhea resistance in the spleen of piglets infected with *C. perfringens* type C, facilitate a better understanding of the resistance mechanism to *C. perfringens* type C in piglets, and provide a reference to improve the ability of piglets to resist diarrhea and breed diarrhea-resistant pig strains. 

## 2. Materials and Methods

### 2.1. Ethical Statement

Piglet experiments were performed in accordance with the regulations for the Administration of Affairs Concerning Experimental Animals (Ministry of Science and Technology, China; revised in June 2004). Sample collection was approved by the ethics committee of Gansu Agricultural University. The animals were euthanized.

### 2.2. C. perfringens Strain Infection and Sample Collection

The test subjects were 30 healthy 7-day-old piglets (Landrace × Yorkshire) (Dingxi Xitai Breeding Co., Ltd., Dingxi, China) and their feces tested negative for *Escherichia coli* F18, *Salmonella*, and *C. perfringens* by enzyme-linked immunosorbent assay. Twenty-five piglets were randomly selected to be challenged, and each piglet was inoculated by oral gavage with 1 mL of 1 × 10^9^ CFU per mL *C. perfringens* type C medium [18]. The remaining five piglets were used as the control group (SC), and those piglets were orally sham-inoculated with sterile culture medium. The trial period was five days. After challenge, the challenged piglets and control piglets were fed in a single stall and an enclosure with the same environmental control and strict disinfection, and they were fed by a specially assigned person. All piglets were fed the same feed and were free to eat and drink. The diarrhea frequency and fecal characteristics of all piglets were recorded daily. Fecal consistency was scored on the basis of visual symptom traits [19]. Then, the 25 challenged piglets were ranked from high to low on the basis of the total diarrhea scores. The top five piglets were the susceptible group (SS) and the bottom five piglets were the resistant group (SR). At the end of the experiment, the experimental piglets were euthanized through inhaled CO_2_. The piglets are placed in an enclosed container and CO_2_ flow was initiated at the minimum rate and time with a constant supply of 80% to 90% CO_2_ to reach a level sufficient to achieve euthanasia. After euthanasia, the spleen tissues of 15 piglets were collected and frozen in liquid nitrogen and stored at −80 °C until RNA extraction.

### 2.3. Total RNA Extraction

Total RNA was extracted using TRIzol reagent (TaKaRa, Dalian, China) according to the manufacturer’s instructions. The quality, purity, and integrity of the total RNA were checked using agar-gel electrophoresis, a Nanodrop 2000 nucleic acid protein detector (Synthgene, Nanjing, China), and an Agilent 2100 analyzer (Agilent, Santa Clara, CA, USA), respectively.

### 2.4. Library Preparation

After a sample was qualified, a Small RNA Sample Pre Kit was used to construct the library. First, a connector was added to both ends of the small RNAs (sRNA). Then, the cDNA was biosynthesized by reverse transcription. After PCR amplification, polyacrylamide gel electrophoresis was used to separate the target DNA fragments, and the cDNA library was obtained by cutting from the gel. Single-ended sequencing of the 15 piglet spleen cDNA sequencing libraries was performed using the Illumina HiSeq 4000 platform (Illumina, San Diego, CA, USA), and 50 bp single-ended sequences were obtained.

### 2.5. Sequencing Data Analysis

First, raw reads were processed through custom Perl and Python scripts. The clean reads were obtained by removing low-quality reads and reads containing poly-N, with 5′ adapter contaminants, without the 3′ adapter or the insert tag, or containing poly A, T, G, or C from the raw data. The Q20, Q30, and GC content of the raw data were evaluated. Then, a certain range of length from the clean reads was chosen to perform all the downstream analyses. Last, the miRNA tags were mapped to the pig reference genome (*Sus scrofa 10.2*) using Bowtie [20].

The mapped small RNA tags were used to search for known miRNAs. The sequences were aligned against the known miRNA precursors and the mature miRNAs deposited in the miRBase 20.0 to identify conserved miRNAs. The characteristics of the hairpin structure of miRNA precursors can be used to predict novel miRNAs. The available software miREvo [21] and miRDeep2 [22] were integrated to predict novel miRNAs through exploring the secondary structure, the Dicer cleavage site, and the minimum free energy of the small RNA tags that were unannotated in the former steps.

When the above sRNAs were compared with all types of annotated RNAs in the pig reference genome, one sRNA could be simultaneously compared with multiple annotation information. Therefore, to ensure that there was only one annotation per sRNA, sRNAs were annotated according to the priority order of known miRNA > rRNA > tRNA > snRNA > snoRNA > repeat > gene > novel miRNA.

### 2.6. Quantification and Differential Expression of miRNAs

The expression levels of miRNAs were counted and normalized using TPM [23]: Normalized expression = (read count × 1,000,000)/libsize (libsize: sum of sample miRNA read count). In samples with biological duplicates, the DESeq R software package, version 4.0 [24] was used to analyze the miRNA differences in the normalized standardized results, and the screening condition for the differential expression of miRNAs was *p*-adjust < 0.05.

### 2.7. Target Gene Prediction and GO and KEGG Enrichment Analysis

The target genes of miRNAs were predicted using miRanda, RNAhybrid, and PITA. Then, the three intersections indicated the candidate target genes of the miRNAs. GOseq [25] and KOBAS software [26] were used to analyze the GO terms and the KEGG signal pathways to understand the functions of the differentially expressed miRNAs.

### 2.8. Screening of miRNAs Correlated with Diarrhea Resistance

Compared with the SC group, the differentially expressed miRNAs between the SR and SS groups were considered to be potential diarrhea resistance-related miRNAs. To separate diarrhea-resistant miRNAs from potential diarrhea-resistant miRNAs, the following screening process was used in this study. First, novel miRNAs that were not annotated in the relevant databases were filtered out. Second, miRNAs without target genes were filtered out. Simultaneously, miRNAs needed to be present in multiple species and with sequences that were highly conserved. Then, the target genes of the selected miRNAs had to be associated with infectious diseases, immune diseases, inflammatory reactions, or similar categories. Last, the target genes of the selected miRNAs had to be enriched in immune response-related KEGG signaling pathways.

### 2.9. Real-Time Quantitative PCR

To validate the data’s accuracy and detect expression of target mRNAs, reverse-transcription quantitative PCR (RT-qPCR) was conducted. All primers for the miRNAs, potential target mRNAs, and the housekeeping gene were designed using the National Center for Biotechnology Information (Table 1 and Table 2). Total RNA was reverse-transcribed to cDNA using a Mir-X™ miRNA FirstStrand Synthesis Kit and a PrimeScript™ RT Reagent Kit with gDNA Eraser (Takara, Dalian, China). The RT-qPCR was performed using TB Green™ Premix Ex Taq™ II (Tli RNaseH Plus) (Takara, Dalian, China). A LightCycler480II Real-Time PCR System (Roche, Basel, Switzerland) was used with the following standard cycling program: initial activation denaturation (95 °C for 3 min), followed by 40 cycles (95 °C for 15 s, 60 °C ± 1 °C for 15 s, and 72 °C for 20 s). miRNA expression and gene expression were quantified relative to U6 and β-actin expression, respectively, using the 2^−∆∆Ct^ method [27]. 

### 2.10. Cell Culture

HEK293T cells were cultured at 37 °C in a humidified incubator with 5% CO_2_ in 25 cm^2^ cell culture flasks (Kirgen, Shanghai, China). The cells were grown in DMEM/F12 medium (HyClone, Logan, UT, USA) supplemented with 10% heat-inactivated fetal bovine serum (Gibco, Waltham, MA, USA) supplemented with 1% penicillin-streptomycin solution (HyClone, Logan, UT, USA).

### 2.11. Dual-Luciferase Reporter Assay

The wild-type or mutant 3′ UTR of *NFATC4* mRNA, containing the miR-133b and miR-532-3p seed sequence targeting sites, was biosynthesized (Genewiz, Suzhou, China) and modified to include *Xho I* and *Sal I* restriction enzyme sites. Subsequently, all the obtained sequences were treated with restriction enzymes, *Xho I* and *Sal I*, and ligated to the pmirGLO dual-luciferase reporter vector (Promega, Madison, AL, USA) using T4 DNA ligase (TaKaRa, Dalian, China). The plasmids were sequenced to verify the correct insertion (Genewiz, Suzhou, China). Lipofectamine 2000 (Invitrogen, Carlsbad, CA, USA) was used to co-transfect HEK293T cells with the wild-type or mutant 3′ UTR luciferase reporter plasmids, and the mimics NC, miR-133b mimic, and miR-532-3p mimic (RiboBio Co., Guangzhou, China). The cells were harvested 48 h after transfection, and the luciferase activities were measured using a Dual-Glo Luciferase Assay System (Promega; Madison, WI, USA). Firefly luciferase was used as the normalization control.

## 3. Results

### 3.1. Total RNA Extraction and Inspection

**Figure 1:**
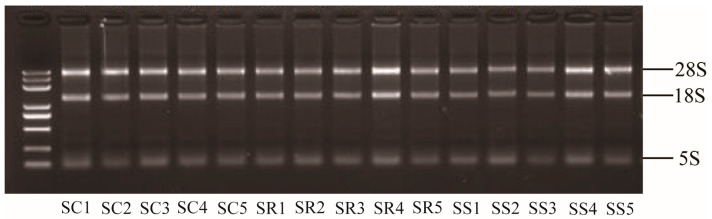
Agarose gel electrophoresis of total RNA of the spleen tissue.

After 1% agarose gel electrophoresis testing, three electrophoresis bands of 28S, 18S, and 5S were clearly visible (Figure 1), indicating that total RNA was not degraded or contaminated. The OD260/280 values ranged from 1.836 to 1.995, the RIN values ranged from 8.0 to 10.0, and the 28S/18S ratio ranged from 1.5 to 2.3 (Table 3), indicating that the RNA samples had good purity and integrity.

### 3.2. Overview of Sequencing Data

A total of 58,170,869, raw reads were obtained for the SC group, 68,439,517 for the SR group, and 64,752,651 for the SS group (Table S1, see Supplementary Materials). After processing the raw reads, a total of 56,404,073 clear reads were obtained from the SC libraries, 66,781,568 from the SR libraries, and 62,854,425 from the SS libraries, accounting for 96.96%, 97.54%, and 97.06% of the raw reads, respectively (Table S1, see Supplementary Materials). Ultimately, clean reads ranging in size from 18 to 35 nt were obtained, with a total of 55,222,330 from the SC libraries, 66,129,541 from the SR libraries, and 60,792,735 from the SS libraries (Table S2, see Supplementary Materials). Lastly, reads totaling 50,663,869 (SC), 61,586,658 (SR), and 55,701,871 (SS) were perfectly mapped to the pig reference genome (Table S2, see Supplementary Materials).

**Figure 2:**
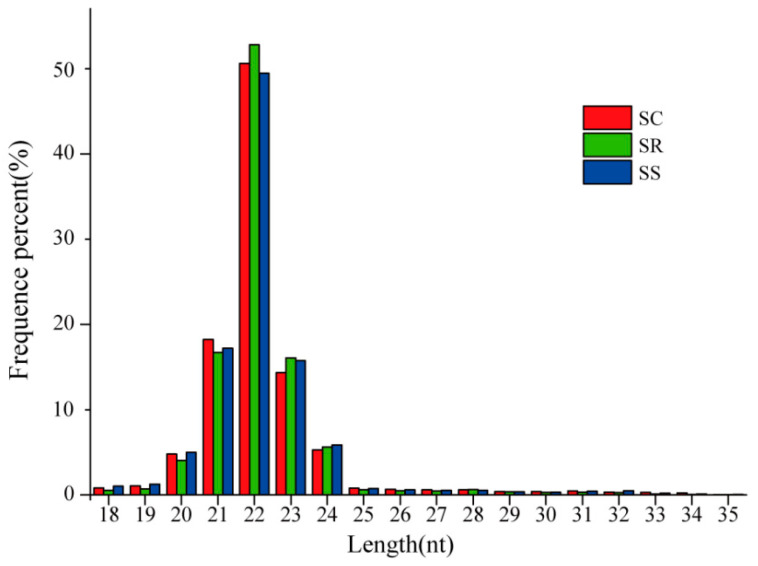
Length distribution of the sequences in SC, SR, and SS libraries.

The lengths of most small RNAs were from 20 to 24 nt, and the largest class contained those with 22 nt, which averaged 50.61% of the total in the SC group, 52.81% in the SR group, and 49.46% in the SS group. The 22 nt class was followed by those with 21 nt (18.23% (SC), 16.69% (SR), and 17.20% (SS)), 23 nt (14.37% (SC), 16.06%(SR), and 15.76%(SS)), and 24 nt (5.29% (SC), 5.61%(SR), and 5.86%(SS)) (Figure 2, Table S3, see Supplementary Materials). The size distribution is typical of sRNA Dicer-processed products.

As shown in Table S4 (see Supplementary Materials), conserved miRNAs (novel miRNAs and known miRNAs) accounted for 49.54% of the total clean reads in the SC sRNA libraries, 54.50% in the SR sRNA libraries, and 49.44% in the SS sRNA libraries. Additionally, conserved miRNAs (novel miRNAs and known miRNAs) accounted for 1.49% of the unique reads in the SC sRNA libraries, 1.69% in the SR sRNA libraries, and 1.38% in the SS sRNA libraries (Table S4, see Supplementary Materials). Most clean reads were classified as miRNA, suggesting that the sequencing was successful in this paper.

### 3.3. Identification of Differentially Expressed miRNAs in Piglet Spleen

**Figure 3:**
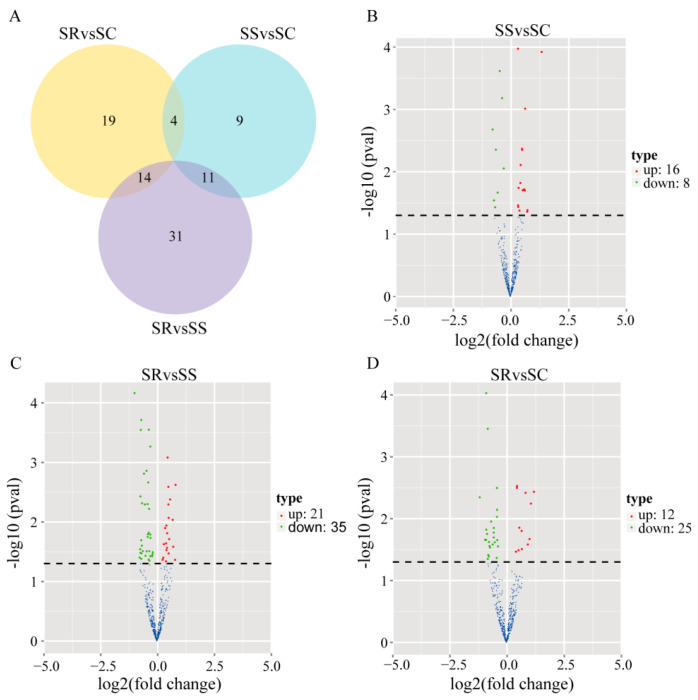
Venn diagram of differentially expressed miRNAs in three comparison groups (**A**). Volcano plot of the differentially expressed miRNAs in SS vs. SC (**B**), SR vs. SS (**C**), and SR vs. SC (**D**).

**Figure 4:**
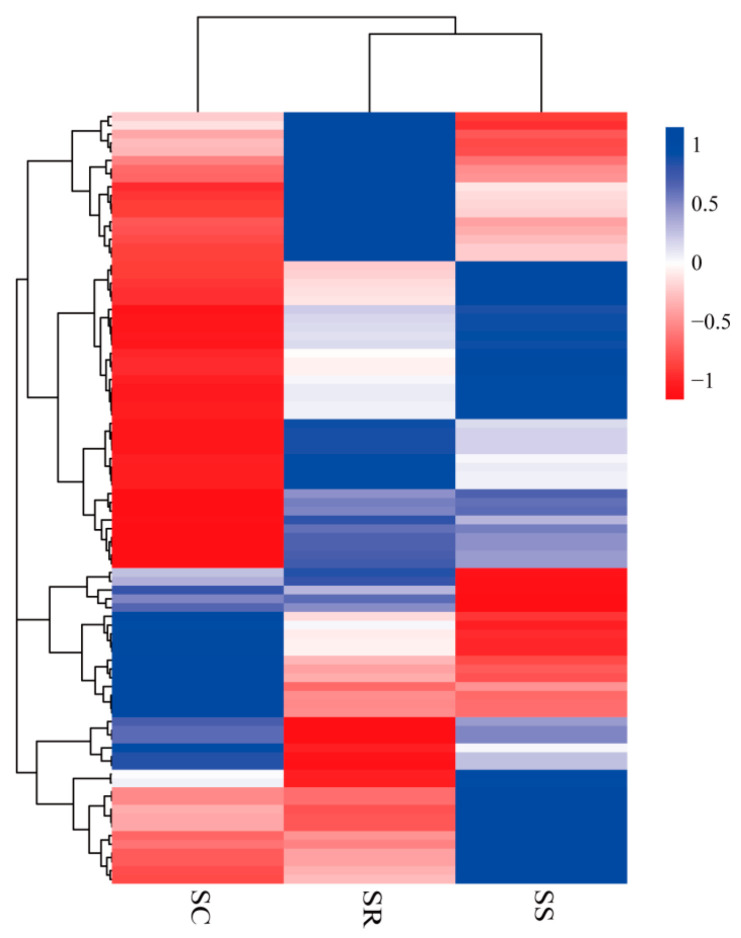
Heat map of the differentially expressed miRNAs in SC, SR, and SS groups.

After successive filtering of these data sets, a total of 852 miRNAs (514 novel miRNAs and 338 known miRNAs) (Table S5, see Supplementary Materials) were identified in the libraries. Among these miRNAs, 88 were differentially expressed with (Figure 3A) 37 (12 up-regulated and 25 down-regulated) in the comparison of the SR and SC groups, 24 (16 up-regulated and 8 down-regulated) in the comparison of the SS and SC groups, and 56 (21 up-regulated and 35 down-regulated) the comparison of the SR and SS groups (Figure 3B–D). Furthermore, the DE miRNAs were used in a systematic cluster analysis to analyze their similarities. The heat map revealed that the SR and SS groups were clustered together because of their similar expression profiles (Figure 4).

### 3.4. Validation of miRNA Expression

**Figure 5:**
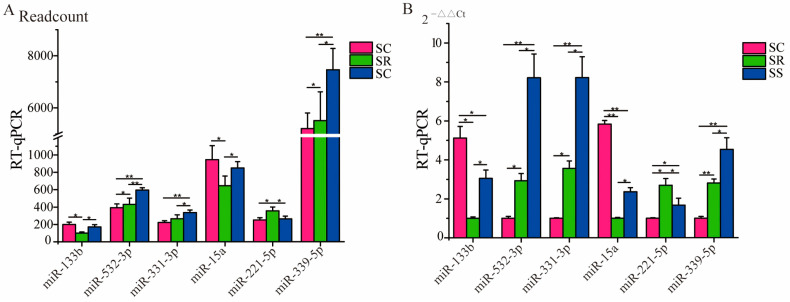
Validation for the RNA sequencing data by qRT-PCR. (**A**) RNA-seq results; (**B**) qRT-PCR results. The results are presented as the mean ± SE. An asterisk denotes a significant difference (* *p* < 0.05; ** *p* < 0.01).

The high-throughput sequencing results (Figure 5A) were generally consistent with the RT-qPCR results (Figure 5B), indicating that the high-throughput sequencing results were accurate and reliable.

### 3.5. miRNA Target Gene Prediction, GO Enrichment, and KEGG Pathway Analyses

A total of 357 target genes were predicted for the 88 differentially expressed miRNAs (Table S6, see Supplementary Materials).

In the comparison of the SS and SC groups, the differentially expressed miRNAs were enriched in 108 GO terms (*p* < 0.01), including 27 in molecular function (MF), 3 in cell composition (CC), and 78 in biological processes (BP) (Table S7, see Supplementary Materials). In the comparison of the SR and SC groups, the differentially expressed miRNAs were enriched in 107 GO terms (*p* < 0.01), including 12 in MF, 20 in CC, and 75 in BP (Table S7, see Supplementary Materials). In the comparison of the SR and SS groups, the differentially expressed miRNAs were enriched in 139 GO terms (*p* < 0.01), including 16 in MF, 27 in CC, and 96 in BP (Table S7, see Supplementary Materials), in which membrane-bounded organelle was significantly enriched (corrected *p* < 0.05).

**Figure 6:**
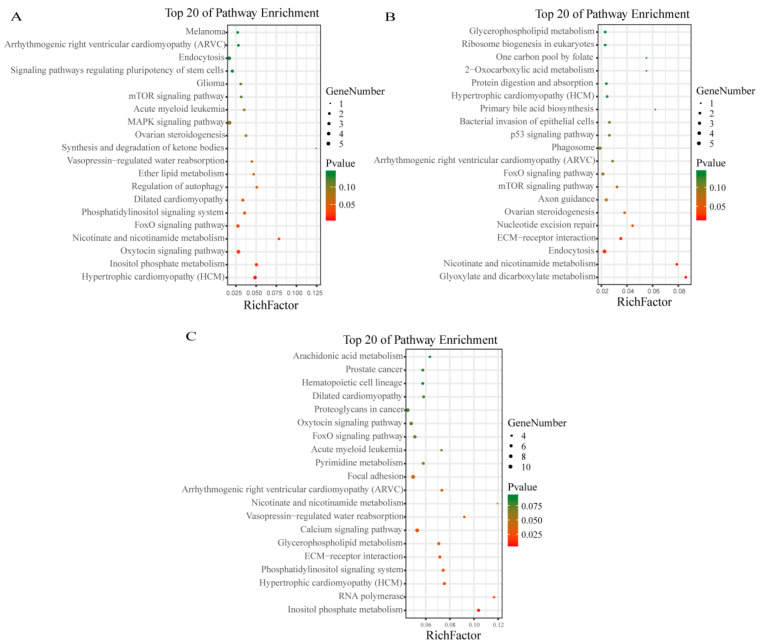
KEGG pathways of differentially expressed miRNA target genes in SS vs. SC (**A**), SR vs. SC (**B**), and SR vs. SS (**C**) groups.

In the comparison of the SS and SC groups, seven significantly enriched signaling pathways were identified; in the comparison of the SR and SC groups, five significantly enriched signaling pathways were identified; and in the comparison of the SR and SS groups, eleven significantly enriched signaling pathways were identified (*p* < 0.05) (Table S8, see Supplementary Materials). Figure 6 shows the top 20 signaling pathways. Some immune-related signaling pathways were identified, such as the MAPK signaling pathway, the p53 signaling pathway, and ECM–receptor interaction.

### 3.6. Screening of Diarrhea Resistance-Associated miRNAs

**Figure 7:**
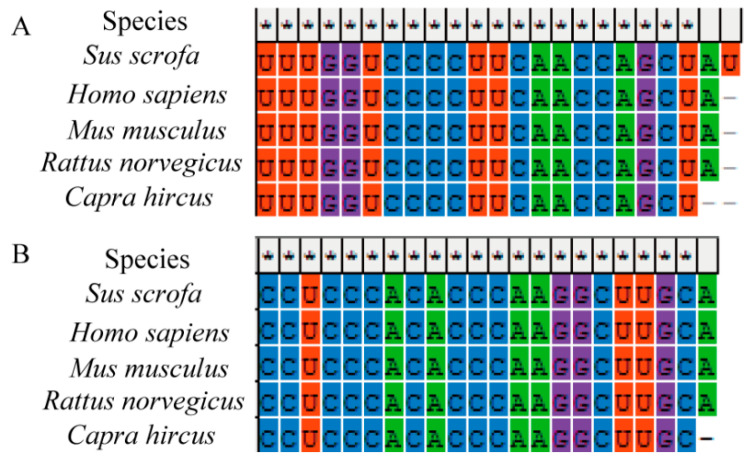
Conservative analysis of miR-133b (**A**) and miR-532-3p (**B**).

Compared with the SC group, 25 miRNAs (6 up-regulated and 19 down-regulated) were differentially expressed in spleen tissues between the SR and SS groups. The information on these miRNAs are displayed in Table 4. Of the 25 miRNAs, 13 had target genes (Table 5). According to the screening criteria of diarrhea resistance-associated miRNAs, four miRNAs (miR-133b, miR-532-3p, miR-339-5p, and miR-331-3p) were ultimately obtained as these were the most closely related to piglet resistance to *C. perfringens* type C. Conservative analysis showed that they were highly conserved and present in multiple species (Figure 7).

### 3.7. Detection of Target Genes Expression of Diarrhea-Resistant miRNAs

**Figure 8:**
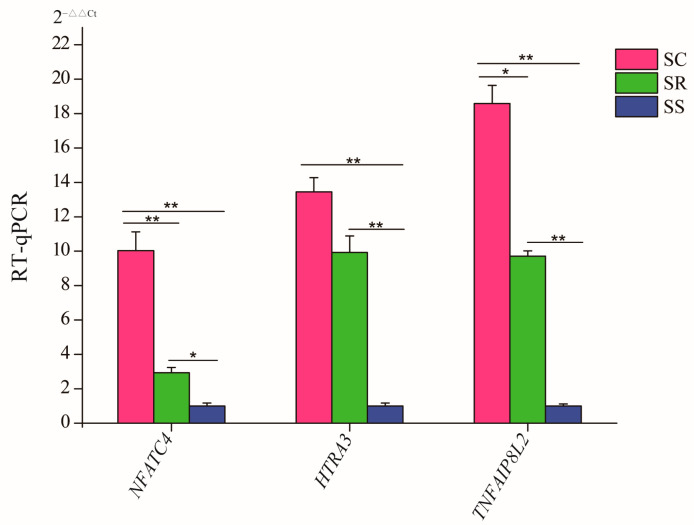
Expression of several potential target mRNAs in SC, SR, and SS groups. The results are presented as the mean ± SE. An asterisk denotes a significant difference (* *p* < 0.05; ** *p* < 0.01).

*NFATC4* was the key target gene of miR-133b and miR-532-3p; *HTRA3* was the key target gene of miR-339-5p; and *TNFAIP8L2* was the key target gene of miR-339-5p and miR-331-3p. These three genes were selected to validate the expression differences in spleen tissues of the SC, SR, and SS groups using RT-qPCR (Figure 8). The changes in the expression of these genes were negative, consistent with the expression of miR-133b and miR-532-3p (*NFATC4*), miR-339-5p (*HTRA3*), and miR-331-3p (*TNFAIP8L2*). The relative expression of *NFATC4*, *HTRA3*, and *TNFAIP8L2* in the SR and SS groups were significantly lower than that in the SC group, and the relative expression of the three genes in the SS group were significantly lower than that in the SR group (*p* < 0.05).

### 3.8. Targeted Regulatory Relationship between miR-532-3p and miR-133b and NFATC4

**Figure 9:**
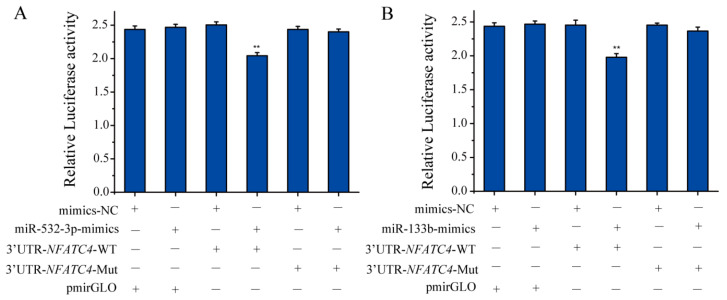
The repressive effect of miR-532-3p (**A**) and miR-133b (**B**) on the activity of *NFATC4* 3′ UTR was measured by luciferase assay. An asterisk denotes a significant difference (** *p* < 0.01).

miR-133b and miR-532-3p play important regulatory roles in suppressing glioma and colorectal cancer through the Wnt/β-catenin signaling pathway, respectively, and *NFATC4* resists *C. perfringens* type C infection in piglets through the Wnt signaling pathway. Therefore, miR-133b and miR-532-3p may participate in resisting piglet *C. perfringens* type C infection through targeted regulation of the expression of *NFATC4*. To confirm the target relationship between miR-532-3p and miR-133b and *NFATC4*, a dual-luciferase reporter assay was performed. The luciferase activities of *NFATC4* decreased significantly after transfection with miR-532-3p mimics and miR-133b mimics. However, no changes in luciferase activity were observed with the plasmids containing the mutated *NFATC4* 3′ UTR (Figure 9). The results suggested that *NFATC4* was a direct target of miR-532-3p and miR-133b.

## 4. Discussion

In past decades, high-throughput sequencing was used to study the expression profiles of miRNAs in various tissues. In this study, a total of 56,404,073 clear reads were obtained from the SC libraries, 66,781,568 from the SR libraries, and 62,854,425 from the SS libraries, which accounted for 96.96%, 97.54%, and 97.06% of the raw reads, respectively (Table S1). In the spleen, 251 novel miRNAs and 317 known miRNAs were discovered (Table S5). To understand the roles of the differentially expressed miRNAs in *C. perfringens* type C infected piglets, the differentially expressed miRNA target genes in the spleen of piglets were analyzed by functional enrichment. Some immune-related signaling pathways, such as the MAPK, p53, and the ECM–receptor interaction signaling pathways, were enriched in the SS and SR groups, compared with the SC group. These pathways were the same as those enriched in spleens in *C. perfringens* type A-infected chickens [8], indicating that the DE miRNAs targeted genes participating in those signaling pathways to resist *C. perfringens* type C infection.

The susceptibility and resistance of piglets to *C. perfringens* type C depends on the genetic makeup of the piglets themselves. To identify the miRNAs that were closely associated with the resistance to *C. perfringens* type C in the spleen of piglets, the differential expression of miRNAs and their target genes was analyzed in the comparison of the SR and SS groups. This analysis was conducted to help understand how these miRNAs played an important regulatory role in piglet resistance to *C. perfringens* type C infection. Compared with the SC group, four miRNAs, miR-133b, miR-532-3p, miR-339-5p, and miR-331-3p, were identified in the SR and SS groups.

These miRNAs play vital roles in cancer development. Lv et al. found that miR-133b suppressed colorectal cancer cell stemness and chemoresistance by targeting the methyltransferase *DOT1L* [28]. Wang et al. found that miR-133b suppressed metastasis by targeting *HOXA9* in human colorectal cancer [29]. Jiang et al. found a suppressive function of miR-532-3p in non-small cell lung cancer (*NSCLC*) by targeting *FOXP3*, revealing that the miR-532-3p/*FOXP3* axis might be a potential therapeutic target for the treatment of *NSCLC* [30]. Zhou et al. found that miR-339-5p regulated the growth, colony formation, and metastasis of colorectal cancer cells by targeting *PRL-1* [31]. Zhao et al. found that miR-331-3p inhibited proliferation and promoted apoptosis by targeting *HER2* through the PI3K/Akt and ERK1/2 pathways in colorectal cancer [32]. These studies showed that miR-133b, miR-532-3p, miR-339-5p, and miR-331-3p were closely associated with piglet resistance to *C. perfringens* type C. All four miRNAs were differentially expressed in the SR and SS groups, were conserved among multiple species, and their target genes were related to the immune response, inflammatory response, and infectious diseases. Some studies suggest that *HTRA3* functions as a pro-apoptotic protein and as a tumor suppressor in the pathogenesis of cancer [33,34]. *HTRA3* is also down-regulated in endometrial, ovarian, and lung cancers [35,36,37,38,39] and upregulated in thyroid tumors [40], suggesting that *HTRA3* is involved in the pathogenesis of cancers. In the current study, the expression of the miR-339-5p target gene *HTRA3* was down-regulated in the SR and SS groups compared to the SC group, although it was particularly higher in the SS group, after the piglets were infected with *C. perfringens* type C. This result indicated that the down-regulation of *HTRA3* could increase the piglets’ immunity and resist the infection of *C. perfringens* type C. *TNFAIP8L2* is a member of the tumor necrosis factor–alpha-induced protein 8 (TNFAIP8) family [41,42]. A newly discovered anti-inflammatory molecule, *TNFAIP8L2*, plays an important regulatory role in suppressing the inflammatory response and maintaining the balance of the immune system [43,44]. In the current study, the expression of the target gene *TNFAIP8L2* of miR-339-5p and miR-331-3p was down-regulated in the SR and SS groups compared with that in the SC group, although it was particularly higher in the SS group after piglets were infected with *C. perfringens* type C. This result indicated that *C. perfringens* type C infection activated immune and inflammatory responses, and that *TNFAIP8L2* helped the piglets resist the inflammatory response caused by *C. perfringens* type C. Nuclear factor of activated T cells (*NFAT*) is a family of transcription factors (*NFATC1-C4*) originally found in activated T lymphocytes [45]. An important gene in the inflammatory response is *NFATC4*. After the inflammatory response, the expression of *NFATC4* is down-regulated, thus activating downstream molecules to participate in inflammation [46,47]. In the current study, the expression of the target gene *NFATC4* of miR-133b and miR-532-3p was down-regulated in the SR and SS groups compared with that in the SC group, although it was particularly higher in the SS group after the piglets were infected *C. perfringens* type C. This result indicated that down-regulation of *NFATC4* was conducive to increasing the degree of immune response and inflammatory response in piglets, so as to resist the *C. perfringens* type C infection.

According to a previous study, miR-7134-5p targeted to *NFATC4* is likely involved to piglet resistance to *C. perfringens* type C through the Wnt signaling pathway [17]. In addition, Xu et al. showed that miR-133b could alleviate glioma development via repressing the Wnt/β-catenin signaling pathway by inhibiting *EZH2*, which provides a potential treatment biomarker for glioma [48]. Gu et al. indicate that miR-532-3p could be a potential candidate for molecular therapy in colorectal cancer through inactivation of the canonical Wnt/β-catenin signaling and enhancement of chemosensitivity [49]. Therefore, the targeting relationships between miR-133b and miR-532-3p and their target gene *NFATC4* were analyzed using a dual-luciferase reporter assay. The luciferase activities of *NFATC4* significantly decreased after transfection with miR-532-3p mimics and miR-133b mimics. However, no change was observed in the luciferase activity of the plasmids containing the mutated *NFATC4* 3′ UTR (Figure 8). These results suggested that *NFATC4* was a direct target of miR-532-3p and miR-133b. miR-133b and miR-532-3p targeted to *NFATC4* were likely involved in piglet resistance to *C. perfringens* type C. This paper provides valuable resources to deeply understand the genetic basis of *C. perfringens* type C resistance in piglets.

## 5. Conclusions

Based on the expression correlation and dual-luciferase reporter assay between the miRNAs and their target genes, we speculate that *NFATC4*, the potential target gene of miR-133b and miR-532-3p, probably acts as a novel marker of *C. perfringens* type C resistance. The present study provides improved database information on pig miRNAs, a better understanding of the genetic basis of *C. perfringens* type C resistance in piglets, and lays a new foundation for identifying novel markers of *C. perfringens* type C resistance.

## Figures and Tables

**Table 1:** miRNA primers for quantitative real-time PCR.

miRNA	miRNA Sequence (5′–3′)	Forward Primer Sequence (5′–3′)
miR-133b	UUUGGUCCCCUUCAACCAGCUAU	TTGGTCCCCTTCAACCAGCTAT
miR-532-3p	CCUCCCACACCCAAGGCUUGCA	TCCCACACCCAAGGCTTGGA
miR-339-5p	UCCCUGUCCUCCAGGAGCUCAC	TGTCCTCCAGGACGCTCAC
miR-331-3p	GCCCCUGGGCCUAUCCUAGAA	CCTGGGCCTATCCTAGAA
miR-15a	UAGCAGCACAUAAUGGUUUGU	CGCAGCACATAATGGTTTGT
miR-221-5p	ACCUGGCAUACAAUGUAGAUUUCUGU	GCACCTGGCATACAATGTAGA

**Table 2:** Oligonucleotide primers used for quantitative real-time PCR.

Gene Name		Primer Sequence (5′–3′)	Product Size (bp)	Accession No.
*HTRA3*	F	GGCCAGTAACCCAGACTTCC	218	NM_001195343.1
	R	TGAAGAGCAGGTCGTCGTTG		
*NFATC4*	F	CTCTGCCCAGCCAGTATGAG	226	XM_013978172.2
	R	ACCTGGTAAAAGGCATGGGG		
*TNFAIP8L2*	F	CTTGGCGAAATGGCTGACTG	208	NM_001204370.1
	R	CTGTTTCCTGTCTTGGGGCT		
*β-actin*	F	TACACCGCTACCAGTTCGCC	218	XM_003124280
	R	GGTCAGGATGCCTCTCTTGC		

**Table 3:** The detection results of total RNA quality.

Sample Name	OD260/280	OD260/230	28S/18S	RIN	Detection Result
SC1	1.968	2.292	1.8	9.7	A
SC2	1.984	2.314	2.1	10.0	A
SC3	1.961	2.291	2.2	9.9	A
SC4	1.931	1.845	2.3	8.5	A
SC5	1.999	1.996	2.1	10.0	A
SR1	1.980	2.062	1.6	8.8	A
SR2	1.951	2.206	2.4	9.4	A
SR3	1.941	2.006	1.8	9.6	A
SR4	1.952	2.092	1.6	8.7	A
SR5	1.959	2.135	2.3	8.8	A
SS1	1.869	2.196	2.0	9.4	A
SS2	1.875	2.462	2.2	9.3	A
SS3	1.968	2.214	1.8	9.4	A
SS4	1.947	2.225	1.8	9.6	A
SS5	1.846	2.246	1.6	9.3	A

**Table 4:** The information of potential diarrheic resistance miRNAs.

miRNA	SR_Readcount	SS_Readcount	*p*
miR-345-5p	86.31	193.83	0.0001
miR-4334-3p	820.64	1436.28	0.0003
miR-107	3563.97	4487.48	0.0006
miR-497	287.88	411.33	0.0015
miR-126-5p	2442.44	3300.18	0.0023
miR-206	340.75	183.73	0.0026
miR-133b	100.27	171.53	0.0052
miR-532-3p	430.9	595.43	0.0054
miR-29c	319.29	488.34	0.0055
miR-125b	19,627.38	25,753.45	0.0065
miR-10a-5p	57,119.99	40,149.33	0.0092
miR-7134-3p	4880.23	3717.64	0.0123
miR-221-5p	355.37	262.24	0.0166
miR-339-5p	5506.21	7453.94	0.0170
miR-7144-5p	30.22	16.74	0.0280
miR-144	693.47	1451.63	0.0307
miR-15a	644.77	847.80	0.0333
miR-18b	18.92	37.17	0.0361
miR-27a	10,048.06	11,849.06	0.0376
miR-16	11,041.7	13,177.6	0.039
miR-331-3p	265.12	336.41	0.0403
miR-4332	1.13	4.21	0.0424
novel-611	12.12	24.72	0.0447
miR-378b-3p	2122.43	2852.25	0.0460
miR-1839-3p	2.13	0.39	0.0464

**Table 5:** The target genes of potential diarrheic resistance-associated miRNAs.

miRNA	Target Genes
miR-133b	*NFATC4*, *AGRP*, *FAM167B*, *ZSWIM4*, *MAFF*
miR-532-3p	*NFATC4*, *GSTZ1*, *FAM167B*, *NELFCD*, *PRADC1*, *IQSEC2*, *HSD17B10*, *ZSWIM4*, *TMEM174*, *MRC2*, *PLCD3*, *SLC4A1*, *SRRM2*, *EDARADD*
miR-339-5p	*KRT82*, *IGF1*, *PARP6*, *KLHL21*, *PQLC2*, *DMRTB1*, *HYI*, *MYO1E*, *DPT*, *KIRREL*, *TNFAIP8L2*, *HTRA3*, *GADD45G*, *BMP1*, *ULK1*, *KIAA1671*, *GATSL3*, *DPYSL4*, *ARHGAP36*, *L1CAM*, *NAT10*, *LAMTOR*, *CEP164*, *HEPACAM*, *TMCC2*, *DDX56*, *TNIP1*, *TMEM92*, *HPCAL4*, *SLC2A6*, *ATAT1*, *SULT1C3*, *MRPS18B*
miR-331-3p	*CSDC2*, *POLR3H*, *PRKAG1*, *KRT82*, *ZNF385A*, *CD27*, *DEF6*, *TEAD3*, *PIM1*, *TREML-2*, *LPCAT4*, *PLEKHG3*, *HSF4*, *LRFN3*, *CADM4*, *PQLC2*, *HEBP2*, *AEN*, *FAM78B*, *NR1I3*, *SLAMF9*, *SHC1*, *TNFAIP8L2*, *STX4*, *ROGDI*, *MCRIP2*, *TMEM129*, *BMP1*, *TBX3*, *DPYSL4*, *COQ8A*, *IL11RA*, *IL17RC*, *PRPS2*, *CDK16*, *ARHGEF9*, *SNX12*, *SLC25A14*, *AVPR2*, *EPS8L2*, *DPF2*, *SPI1*, *ABTB2*, *PTPN5*, *NCLN*, *MARCH2*, *ATG16L2*, *IL10RA*, *FGFR1*, *TTYH2*, *MRC2*, *HCRT*, *IGFBP4*, *CACNB1*, *TMEM92*, *RAB11FIP4*, *SOX15*, *NT5M*, *RIMS4*, *OXTR*, *SLC46A1*, *ZNF235*, *MFSD7*, *BPIFB3*, *SLC23A1*, *PLA2G16*, *TMCO4*, *SLC22A7*, *GAL3ST3*, *FBXO18*, *SBNO2*, *GANAB*
miR-345-5p	*TIMM22*
miR-4334-3p	*ACO2*, *GRASP*, *KRT82*, *ITGA5*, *IGF*, *TRABD*, *PPARD*, *MDFI*, *PARP6*, *SLC25A29*, *CLEC18A*, *DHX34*, *KLHL21*, *PQLC2*, *DMRTB1*, *LRRC41*, *HYI*, *FRMD1*, *ANGPTL2*, *PTPA*, *TRAPPC9*, *DPT*, *KIRREL*, *TNFAIP8L2*, *NUDT17*, *RALY*, *NNAT*, *ABHD11*, *PKDCC*, *HTRA3*, *CUL4A*, *GADD45G*, *BMP1*, *ULK1*, *KIAA1671*, *GATSL3*, *AIFM3*, *MED15*, *NUP210*, *SYP*, *FOXP3*, *ARHGAP36*, *PDZD4*, *L1CAM*, *DEAF1*, *SNX32*, *TM7SF2*, *AMBRA1*, *SPPL2B*, *CTNNA1*, *LAMTOR1*, *ARAP1*, *CEP164*, *HEPACAM*, *TMCC2*, *CNPPD1*, *DDX56*, *TNIP1*, *TMEM92*, *BCAM*, *CD3EAP*, *POLR2E*, *WDR4*, *HPCAL4*, *NR2C2AP*, *SLC2A6*, *ATAT1*, *FTCD*, *SNRPN*, *FAM207A*, *SULT1C3*, *LBP*, *VPS37C*, *MRPS18B*
miR-497	*SLC3A1*, *TMEM61*, *TTLL5*, *GP1BB*
miR-29c	*EXOSC10*
miR-7144-5p	*MORC2*, *CCR10*
miR-27a	*DMKN*, *MOSPD3*
miR-4332	*HTRA3*, *ALOX5*
novel_611	*GTPBP1*, *FAR2*, *FOXN3*, *AEN*, *SLC39A1*, *SLC7A2*, *TUBB1*, *AIFM3*, *HPS1CDHR5*, *RAB3GAP1*, *COA3*, *KLHDC8A*, *CEP85*
miR-378b-3p	*ECI2*, *CLK2*, *ARAP1*, *GHRHR*, *SLC2A6*

## Data Availability

The sequencing data was obtained from GEO (accession number GSE145302) in NCBI.

## Supplementary Materials

The following supporting information can be downloaded at: https://www.mdpi.com/article/10.3390/cimb45040208/s1, Table S1: Summery of small RNA sequence; Table S2: Genome localization of small RNA; Table S3: Length distribution of the sequences in SC, SR, and SS libraries; Table S4: Classification annotation of total reads and unique reads of small RNAs; Table S5: Differentially expressed miRNAs in SS vs SC, SR vs SC, and SR vs SS; Table S6: Predicted target genes of differentially expressed miRNAs in SS vs SC, SR vs SC, and SR vs SS; Table S7: GO functional enrichment of predicted target genes of differentially expressed miRNAs in SS vs SC, SR vs SC, and SR vs SS; Table S8: KEGG pathway functional enrichment of predicted target genes of differentially expressed miRNAs in SS vs SC, SR vs SC, and SR vs SS.
